# Fluopyram Sensitivity and Functional Characterization of SdhB in the *Fusarium solani* Species Complex Causing Soybean Sudden Death Syndrome

**DOI:** 10.3389/fmicb.2018.02335

**Published:** 2018-10-01

**Authors:** Hyunkyu Sang, Alexander Witte, Janette L. Jacobs, Hao-Xun Chang, Jie Wang, Mitchell G. Roth, Martin I. Chilvers

**Affiliations:** ^1^Department of Plant, Soil and Microbial Sciences, Michigan State University, East Lansing, MI, United States; ^2^Department of Plant Biology, Michigan State University, East Lansing, MI, United States; ^3^Genetics Graduate Program, Michigan State University, East Lansing, MI, United States

**Keywords:** fluopyram, *Fusarium brasiliense*, *Fusarium tucumaniae*, *Fusarium virguliforme*, root rot, SdhB, soybean sudden death syndrome

## Abstract

The succinate dehydrogenase inhibitor (SDHI) fungicide, fluopyram, is used as a soybean seed treatment to manage *Fusarium virguliforme*, the casual agent of sudden death syndrome (SDS). More recently, other species within clade 2 of the *Fusarium solani* species, *F. tucumaniae* in South America and *F. brasiliense* in America and Africa, have been recognized as additional agents capable of causing SDS. To determine if fluopyram could be used for management of SDS caused by these species, *in vitro* sensitivity tests of the three *Fusarium* species to fluopyram were conducted. The mean EC_50_ values of *F. brasiliense* and *F. virguliforme* strains to fluopyram were 1.96 and 2.21 μg ml^-1^, respectively, but interestingly *F. tucumaniae* strains were highly sensitive (mean EC_50_ = 0.25 μg ml^-1^) to fluopyram compared to strains of the other two species. A sequence analysis of *Sdh* genes of *Fusarium* strains revealed that the *F. tucumaniae* strains contain an arginine at codon 277 in the *Sdh*B gene instead of a glycine as in other *Fusarium* species. Replacement of glycine to arginine in SdhB-277 in a *F. virguliforme* wild-type strain Mont-1 through genetic transformation resulted in increased sensitivity to two SDHI fungicides, fluopyram and boscalid. Similar to a *F. tucumaniae* strain, the Mont-1 (SdhB^G277R^) mutant caused less SDS and root rot disease than Mont-1 on soybean seedlings with the fluopyram seed treatment. Our study suggests the amino acid difference in the SdhB in *F. tucumaniae* results in fluopyram being efficacious if used as a seed treatment for management of *F. tucumaniae*, which is the most abundant SDS causing species in South America. The establishment of baseline sensitivity of *Fusarium* species to fluopyram will contribute to effective strategies for managing *Fusarium* diseases in soybean and other pathosystems such as dry bean.

## Introduction

Soybean sudden death syndrome (SDS) caused by *Fusarium virguliforme*, is an economically devastating disease in North America. SDS ranked among the top forth yield-suppressing soybean diseases and 209.7 million bushels of soybean yield loss were attributed to SDS during 2010-2014 in the United States and Ontario, Canada (Allen et al., 2017). SDS outbreaks caused by *F. virguliforme* have been reported in nearly all major soybean producing States of the U.S. except North Dakota (Pennypacker, 1983; Rupe, 1989; Roy, 1997; Roy et al., 1997; Mulrooney et al., 2002; Aoki et al., 2003; Kurle et al., 2003; Ziems et al., 2006; Bernstein et al., 2007; Chilvers and Brown-Rytlewski, 2010). While *F. virguliforme* is the dominant SDS pathogen in North America, SDS outbreaks have predominantly been attributed to *F. tucumaniae* in South America and *F. brasiliense* especially in Brazil (O’Donnell et al., 2010). A recent survey of SDS symptomatic soybean fields in Michigan found that *F. brasiliense* was also present in the U.S. (Wang et al., unpublished). All of these SDS causing species belong to clade 2 of the *Fusarium solani* species complex (FSSC), which also contains species capable of causing root rot, but possibly little to no SDS (O’Donnell et al., 2010).

Due to the significant economic impact of SDS in soybean production, different management strategies such as utilizing partially resistant varieties, crop rotation, tillage and planting date have been investigated to manage SDS (Wrather et al., 1995; Leandro et al., 2018). Soybean seed treatments have been used routinely to protect from early infection by root rot pathogens but most seed treatment fungicides are not effective against SDS (Weems et al., 2015). In 2014, the succinate dehydrogenase inhibitor (SDHI), fluopyram, was registered as a seed treatment (ILeVO, Bayer Crop Science) and has been widely used in soybean fields because of its effective suppression of SDS (Kandel et al., 2016, 2018). Wang et al. (2017) established baseline sensitivity of *F. virguliforme* isolates from different states to fluopyram. The majority of *F. virguliforme* isolates characterized in the study displayed sensitivity to fluopyram (mean EC_50_ = 3.35 μg ml^-1^) (Wang et al., 2017). Although this information is useful in efforts to prolong the product life of fluopyram to *F. virguliforme*, the sensitivity of other important SDS pathogens like *F. brasiliense* and *F. tucumaniae* to fluopyram has not been determined.

The SDHIs are new generation fungicides and have been widely applied to manage many important plant pathogenic fungi (Sierotzki and Scalliet, 2013). Currently, 11 different chemical groups and 23 common name SDHIs are listed by the Fungicide Resistance Action Committee (FRAC, 2018a). These chemicals have a common site-specific mode of action within mitochondria, strongly binding to ubiquinone-binding sites (Qp) in the succinate dehydrogenase complex composed of four subunits (SdhA, SdhB, SdhC, and SdhD). The binding of SDHIs results in blockage of access to the substrate, which consequently prevents further catalyzation of the oxidation of succinate to fumarate and reduction of ubiquinone to quinone. This leads to reduced energy production and arrested fungal growth (Matsson and Hederstedt, 2001; Sierotzki and Scalliet, 2013). Resistance to SDHIs has been reported in 23 plant pathogenic fungi to date and is associated with mutation(s) in *Sdh*B, *Sdh*C, and/or *Sdh*D genes (FRAC, 2015; FRAC, 2017; Popko et al., 2018). Because SDHIs have been widely applied as foliar fungicides, most field-developed SDHI-resistant pathogens (e.g., *Alternaria alternata, Botrytis cinerea, Zymoseptoria tritici*) are air-borne foliar pathogens (Sierotzki and Scalliet, 2013). SDHIs are also used as seed treatments, but knowledge in the exposure of soil-borne pathogens such as *Fusarium* species to SDHIs is limited and it is assumed that risk for development of SDHIs resistance for soil-borne pathogens is considerably less than for foliar pathogens. Despite the importance of soil-borne pathogens in agricultural production, *Fusarium* species such as the SDS casual pathogens have not been extensively studied in terms of SDHI resistance nor the function of their *Sdh* genes related to SDHIs.

In recent years, soybean SDS caused by *F. virguliforme, F. brasiliense*, and *F. tucumaniae* has become a significant problem not only in the United States but also in South America and South Africa (Aoki et al., 2003; Tewoldemedhin et al., 2017; Wang et al., unpublished). To protect soybean yields from losses caused by these SDS pathogens, an understanding of the pathogens’ sensitivity to fungicides and mechanism on target genes is crucial. Fluopyram as a seed treatment currently demonstrates the best field efficacy to manage *F. virguliforme* and the SDS that it causes. To address the concerns outlined above three objectives were established: (1) perform an *in vitro* sensitivity assay of *F. virguliforme, F. brasiliense*, and *F. tucumaniae* strains to fluopyram, (2) compare the sequences of their *Sdh* genes to investigate genetic factor(s) driving fungicide sensitivity, (3) validate the involvement of genetic factor(s) in fluopyram sensitivity using a reverse genetics approach and virulence on fluopyram treated soybean seed.

## Materials and Methods

### *Fungal* Strains

A total of 35 FSSC strains were used in this study. The species (n = number of strains used) are as follows: *F. brasiliense* (*n* = 17), *F. tucumaniae* (*n* = 4), *F. virguliforme* (*n* = 14). Thirteen *Fusarium* strains were obtained from the Agricultural Research Service Culture Collection (NRRL - Northern Regional Research Laboratory, United States). Additional strains were obtained from two different studies (Wang and Chilvers, 2016; Wang et al., unpublished). The origins and hosts are described in **Table 1**.

**Table 1:** *Fusarium* strains used in this study and their sensitivity to fluopyram.

*Fusarium* species	Strains	Hosts	Origins	EC_50_ to fluopyram
*F. brasiliense*	NRRL 22678	*Glycine max*	California, United States	1.06
	MI-Mtc-A3	*Glycine max*	Michigan, United States	1.76
	MI-Mtc-A8	*Glycine max*	Michigan, United States	1.15
	MI-Mtc-A17	*Glycine max*	Michigan, United States	3.31
	MI-Mtc-B1	*Glycine max*	Michigan, United States	0.94
	MI-Mtc-B5	*Glycine max*	Michigan, United States	1.62
	MI-Mtc-B6	*Glycine max*	Michigan, United States	2.41
	MI-Mtc-B9blu	*Glycine max*	Michigan, United States	2.99
	MI-Mtc-B9brn	*Glycine max*	Michigan, United States	4.24
	MI-Mtc-B11	*Glycine max*	Michigan, United States	3.83
	MI-Mtc-B15	*Glycine max*	Michigan, United States	0.80
	MI-Mtc-C1	*Glycine max*	Michigan, United States	0.91
	MI-Mtc-C2	*Glycine max*	Michigan, United States	0.80
	MI-Mtc-C3	*Glycine max*	Michigan, United States	1.06
	MI-Mtc-C6	*Glycine max*	Michigan, United States	2.69
	MI-Mtc-C12	*Glycine max*	Michigan, United States	1.62
	F_14-43	*Phaseolus vulgaris*	Michigan, United States	2.57
*F. tucumaniae*	NRRL 31096	*Glycine max*	San Agustín, Argentina	0.31
	NRRL 31777	*Glycine max*	Rio Grande do Sul, Brazil	0.06
	NRRL 31950	*Glycine max*	Paraná, Brazil	0.24
	NRRL 34549	*Glycine max*	Buenos Aires, Argentina	0.40
*F. virguliforme*	Mont-1 (NRRL 22292)	*Glycine max*	Illinois, United States	2.59
	NRRL 32392	*Glycine max*	Wisconsin, United States	2.99
	NRRL 34553	*Glycine max*	Santa Fe, Argentina	2.17
	NRRL 36607	*Soil*	Buenos Aires, Argentina	2.06
	NRRL 36898	*Glycine max*	Santa Fe, Argentina	2.45
	NRRL 36900	*Glycine max*	Santa Fe, Argentina	2.32
	NRRL 37591	*Glycine max*	Missouri, United States	2.58
	NRRL 54291	*Glycine max*	Buenos Aires, Argentina	1.90
	MISTJ-C6	*Glycine max*	Michigan, United States	2.69
	CL-15-01	*Glycine max*	Michigan, United States	1.87
	MISTJ-A3	*Glycine max*	Michigan, United States	1.32
	MIVB-B5	*Glycine max*	Michigan, United States	1.70
	MO4a	*Glycine max*	Missouri, United States	1.53
	LL0028	*Glycine max*	Iowa, United States	2.34

### *In vitro* Sensitivity Assays of *Fusarium* Strains to Fluopyram

Thirty five strains of *Fusarium* species were grown on half-strength PDA (19.5 g of PDA and 7.5 g of agar in 1 L of distilled water) for 10 days. Half-strength PDA was used to increase the growth rate of *Fusarium* species. Agar plugs from actively growing colonies were inoculated on half-strength PDA without and with 0.5, 1, 3, 5, 7, 10, 50 μg ml^-1^ of fluopyram (Luna Privilege, Bayer CropScience, containing 43% of active ingredient). After 10 days, colonies were imaged using a scanner according to the method described in (Wang et al., 2017). The effective fungicide concentration to reduce mycelial growth rate by 50% (EC_50_) was calculated using R (R Core Team, 2015) package “drc” (Ritz and Streibig, 2005) with the four-parameter log-logistic model (LL.4). Two separate experiments and three replicates (petri plates) of each treatment per each experiment were conducted for each isolate.

### Sequences Analysis of *Sdh*B, *Sdh*C, and *Sdh*D Genes in Fungal Species

To compare the sequences of *Sdh*B, *Sdh*C, and *Sdh*D genes of three *Fusarium* species, the genes were amplified and sequenced from DNA of seven *F. brasiliense* strains (NRRL 22678, MI-Mtc-A8, MI-Mtc-A17, MI-Mtc-B6, MI-Mtc-B9brn, MI-Mtc-B11, and MIMtc-C6), four *F. tucumaniae* strains (NRRL 31096, NRRL 31777, NRRL 31950, and NRRL 34549), and five *F. virguliforme* strains (Mont-1, NRRL 32392, NRRL 36898, NRRL 36900, and NRRL 54291). Sensitivities of *F. virguliforme* strains to fluopyram were similar, as such strains were randomly selected for sequencing. Strains of *F. brasiliense* demonstrated a range of sensitivity to fluopyram, as such strains were selected to represent this range for sequence analysis. The genome sequences of *F. virguliforme* (Mont1 (NRRL 22292), NRRL 34551, LL0009, and Clinton-1B), *F. brasiliense* (NRRL 31757), and *F. tucumaniae* (NRRL 31096, NRRL 31781, and NRRL 34546) (GenBank BioProject Accession: PRJNA63281 and PRJNA289542) (Srivastava et al., 2014; Huang et al., 2016) were obtained from NCBI. *Sdh* genes were mined from the genome sequences using BLAST+ (Camacho et al., 2009) and used for the reference sequences and primer design. DNA from the strains were extracted by a CTAB method (Cubero et al., 1999) and each gene was amplified using Phusion High-Fidelity DNA Polymerase mixtures (New England BioLabs) with primer pairs described in **Supplementary Table S1**. The final PCR amplicons were purified by ExoSAP-IT (Affymetrix) and sequenced by the Research Technology Support Facility at Michigan State University. MEGA v6.06 was used for sequencing analysis of the *Sdh* genes (Tamura et al., 2013).

### Generation of *Fusarium virguliforme* SdhB^G277R^ Mutants

The upstream and full length of *Sdh*B (1,126 bp) was amplified from gDNA of *F. virguliforme* strain Mont-1 (NRRL 22292) using primers F_KpnI_FvSdhB and R_SpeI_FtSdhB. The amplified fragment contained 949A, instead of 949G, because the reverse primer was designed for this replacement. The downstream region of SdhB (1,000 bp) was amplified from gDNA of Mont-1 using primers F_NotI_downFvSdhB and R_NsiI_downFvSdhB. Amplicons of the *Sdh*B gene and downstream region of *Sdh*B were digested by restriction enzymes KpnI/SpeI and NotI/NsiI, respectively, and purified by Monarch DNA Gel Extraction Kit (New England BioLabs). Each purified fragment was inserted into Topo-hph plasmid (Sang et al., 2017) digested by KpnI/SpeI or NotI/NsiI to generate Topo-hph-FvSdhB(G277R) or Topo-hph-downFvSdhB. Using the two generated plasmids as a DNA template, two DNA constructs were amplified with primers F_FvSdhB/R_YG and F_HY/R_downFvSdhB, respectively, each containing 1,126 bp of the upstream and full length of *Sdh*B and 731 bp of hph or 1 kb downstream of the *Sdh*B and 1,126 bp of PtrpC-hph. The constructs were gel-purified using the Gel Extraction Kit, and the final two DNA constructs (each 2 μg) were transformed using a polyethylene glycol (PEG)-mediated transformation method in protoplasts from the *F. virguliforme* strain Mont-1. The protoplast generation and PEG-mediated transformation were conducted using a modified method from Mansouri et al. (2009) and Hallen-Adams et al. (2011). After the generation of hygromycin resistant *F. virguliforme* transformants, single spore isolation of each transformant was conducted and DNA of the transformants was extracted using a CTAB method (Cubero et al., 1999). The four primer sets (F_detFvSdhB/R_detFvSdhB; F_detFvSdhB/R_YG; F_HY/R_detFvSdhB; F_ptrpC/R_hgh) were used to confirm Mont-1(SdhB^G277R^) mutants and the fragment amplified by the primer pair (F_detFvSdhB/R_detFvSdhB) was sequenced.

### Sensitivity Assays of the *F. virguliforme* Mutant Strains to SDHI Fungicides

To investigate the effect of a G277R substitution in the *Sdh*B gene of the strain Mont-1, sensitivity of *F. virguliforme* mutants Mont-1(SdhB^G277R^)-1 and Mont1(SdhB^G277R^)-2 to two different SDHI fungicides fluopyram and boscalid was assayed. Boscalid was added in this assay to test whether the mutation in *Sdh*B confers cross sensitivity to two different chemical groups of SDHI fungicides, carboxamide (boscalid) and pyridinylethyl-benzamide (fluopyram). Two *F. virguliforme* strains (Mont-1 and NRRL 54291) and two *F. tucumaniae* strains (NRRL 31096, and NRRL 34549) were included in this sensitivity assay as reference strains. The six strains were grown on PDA for 8 days and the agar plugs (3 mm in diameter) from the edge of a fungal colony were inoculated onto the half-strength PDA without or with fluopyram (1 μg ml^-1^) and boscalid (Endura, BASF, containing 70% of active ingredients; 50 μg ml^-1^). After 5 days, pictures of strains on the half-strength PDA and half-strength PDA amended with fluopyram or boscalid were taken and the diameters of each colony were measured. Two separate experiments and three replicates per each experiment were conducted.

### Soybean Seedling Pathogenicity Assay of *F. virguliforme* and *F. tucumaniae* Strains to the Seed Treatment With Fluopyram

The pathogenicity of *F. virguliforme* strains Mont-1 and Mont-1(SdhB^G277R^)-1 and *F. tucumaniae* NRRL 31096 were assayed on soybean seedlings from two different seed varieties (A and B) without treatment, with base seed treatment, and with base + fluopyram seed treatment. Base seed treatment (Bayer CropScience) contained prothioconazole + penflufen + Metalaxyl (EverGol Energy, 0.019 mg a.i./seed; Bayer CropScience), Metalaxyl (Allegiance, 0.02 mg a.i./seed; Bayer CropScience), and clothianodin + *B. firmus* (Poncho/VOTiVO, 0.13 mg a.i./seed; Bayer CropScience). The base + fluopyram included fluopyram at a rate of 0.15 mg a.i./seed. Pro-Ized red seed colorant (Gustafson LLC) and finisher (Peridiam Precise 1010; Bayer CropScience) were added at the rate of 32.6 and 65 ml/100kg of seed, respectively. Fungal inoculum was prepared by the method used in Wang et al. (2018). Briefly, each strain was grown on Nash-Snyder (NS) medium for 14 days at room temperature. A fungal colonized NS medium plate and a non-colonized NS medium plate, and 100 ml of autoclaved deionized water were mixed and homogenized in a sterile stainless-steel blender carafe for 30 s. The homogenized inoculum slurry was inoculated to autoclaved sorghum grain (1.8 kg) in spawn bags (Fungi perfecti) and allowed to colonize the grain for 4 weeks at room temperature. The grain inoculum in the bag were mixed by shaking every day. For seedling pathogenicity assay, twelve grams of infested grain inoculum or control grain were mixed with 250 ml of medium vermiculite and placed into a 354 ml paper cup (Solo) with three drainage holes on the bottom. Seventy ml of the medium vermiculite was poured over the inoculum-vermiculite mixture in the cup and six soybean seeds were sown in each cup. After that, seeds were covered with an additional 70 ml of the medium vermiculite. The cups were incubated in a growth chamber at 20°C with 14 h light period and watered as needed. After 28 days of incubation, SDS disease severity index was rated (scale 1–9): 1 = healthy plant, 2 = leaf showing slight yellowing and/or chlorotic flecks or blotches, 3 = leaf with interveinal chlorosis, 4 = leaf with necrosis along a portion (>2 cm) of its margin with interveinal chlorosis. 5 = necrosis along the entire margin of leaf with leaf or leaves cupped and/or irregular. 6 = interveinal necrosis and necrotic and/or leaf loss of most of the leaf area, 7 = most of the leaf area is necrotic and entire defoliated plants with new growth, 8 = entirely defoliated plants and most plants dead or dying, 9 = plant dead or dying. Roots were harvested and washed, and root rot disease severity was rated (scale:1–7) according to the modified method described previously by Schneider and Kelly (2000): 1 = healthy roots, 2 = small lesions on tap root or lateral root, 3 = increased size or coalescing of root lesion with 1–10% reduction in root mass, 4 = increased root lesion length with full encircling of root and 10–20% reduction in root mass, 5 = increase in root lesion elongation with tap root rotted and 20–50% reduction in root mass, 6 = intense root discoloration and root with 50–80% root mass reduction, 7 = pithy or hollow root with portion of root falling off with 80–100% root mass reduction and functionally dead. The washed roots were dried for 2 days at 55°C and dry root and shoot mass (g) were measured. The experiment with two different soybean varieties was performed with three replicate pots (six seeds in each pot).

### Statistical Analysis

All statistical analyses were conducted by the JMP software package, version 14.0 (SAS Institute Inc.).

## Results

### Sensitivity of SDS Causal *Fusarium* Species to Fluopyram

In order to establish the sensitivity of three *Fusarium* species to fluopyram, EC_50_ values were measured using 35 strains from geographically different locations (**Table 1**). The mean EC_50_ values of *F. brasiliense* (*n* = 17), *F. tucumaniae* (*n* = 4), and *F. virguliforme* (*n* = 14) to fluopyram were 1.96 ± 0.2 μg ml^-1^, 0.25 ± 0.1 μg ml^-1^, and 2.21 ± 0.1 μg ml^-1^, respectively. The EC_50_ values among *F. brasiliense, F. virguliforme*, or *F. tucumaniae* strains were not statistically different (*P* = 0.091, *P* = 0.132, and *P* = 0.637, respectively). However, EC_50_ values among all *Fusarium* strains were statistically significant (*P* = 0.0013). The mean EC_50_ value of *F. tucumaniae* was significantly lower than the values of *F. brasiliense* (*P* = 0.0013) or *F. virguliforme* (*P* < 0.0001). There was no statistical difference between the mean EC_50_ values of *F. brasiliense* and *F. virguliforme*. The EC_50_ range of *F. tucumaniae, F. brasiliense*, and *F. virgulforme* strains were 0.06 to 0.4 μg ml^-1^, 0.8 to 4.24 μg ml^-1^, 1.53 to 2.99 μg ml^-1^ (**Table 1**).

### Comparative Analysis of Sequences of *Sdh* Genes From Strains of Three *Fusarium* Species

**FIGURE 1:**
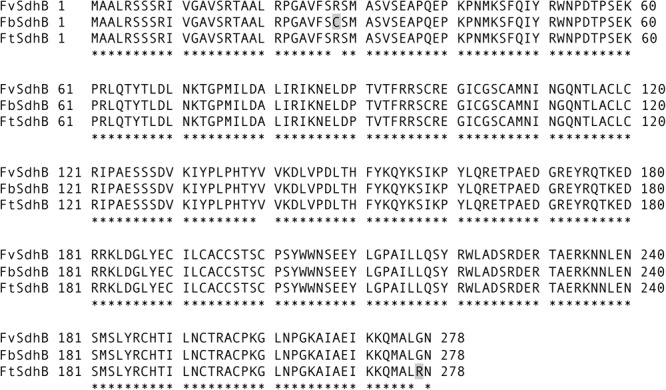
Alignment of the full length of SdhB amino acid sequences of *F. brasiliense, F. virguliforme*, and *F. tucumaniae* strains. FbSdhB, FvSdhB, and FtSdhB refer to SdhB amino acid sequence from *F. brasiliense, F. virguliforme*, and *F. tucumaniae*, respectively. The shaded letters indicate difference at amino acid position 28 in *F. brasiliense* from other species and 277 in *F. tucumaniae* from other species. Asterisks indicate identical amino acids among the strains compared.

To investigate the potential genetic factor(s) driving the sensitivity of *F. brasiliense, F. tucumaniae*, and *F. virguliforme* strains to fluopyram, the SDHI target genes (*Sdh*B, *Sdh*C, and *Sdh*D) of 16 strains (*F. brasiliense* strains: NRRL 22678, MIMtc-A8, MIMtc-A17, MIMtc-B6, MIMtc-B9brn, MIMtc-B11, and MIMtc-C6; *F. tucumaniae* strains: NRRL 31096, NRRL 31777, NRRL 31950, and NRRL 34549; *F. virguliforme* strains: Mont-1, NRRL 32392, NRRL 36898, NRRL 36900, and NRRL 54291) were sequenced and compared with reference sequences from eight genome sequences of *F. brasiliense, F. tucumaniae*, and *F. virguliforme* strains. Amino acid sequences of SdhC and SdhD from *F. brasiliense, F. tucumaniae*, and *F. virgulforme* strains and the *Fusarium* genome sequences were identical, except *Sdh*D sequence from one of genome sequences (NRRL 34546), which has a gap in the coding sequences of *Sdh*D. However, two amino acid differences were detected in SdhB among three *Fusarium* species. Both *F. tucumaniae* and *F. virgulforme* strains have arginine (R) at codon 28 of SdhB but *F. brasiliense* strains (except for one strain, MI-Mtc-B6) contain cysteine (C) at the same position. *F. brasiliense* and *F. virgulforme* strains have glycine (G) at codon 277 of SdhB but *F. tucumaniae* strains contain arginine (R) at the same position (**Figure 1**). All amino acid sequences of SdhB, SdhC, and SdhD from *F. brasiliense, F. tucumaniae*, and *F. virguliforme* strains are presented in **Supplementary Table S2**.

### An Amino Acid Substitution (G277R) in SdhB Causes Increased Sensitivity to SDHI Fungicides in *F. virguliforme*

**FIGURE 2:**
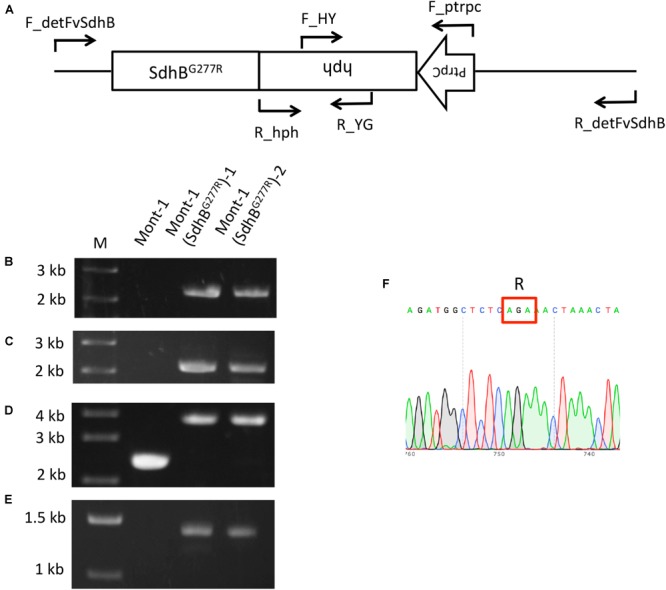
Validation of *F. virguliforme* mutants Mont-1(SdhB^G277R^)-1 and -2. **(A)** Schematic diagram of the *Sdh*B gene and hygromycin resistance cassette (PtrpC and hph) and short arrows indicate primer binding sites. **(B)** Primer pair F_detFvSdhB/R_YG was used to amplify the 2,058-bp fragment of the left flanking region. **(C)** Primer pair F_HY /R_detFvSdhB was used to amplify the 2,171-bp fragment of right flanking region. **(D)** Primer pair F_detFvSdhB/R_detFvSdhB was used to amplify the fragment containing upstream and full length of *Sdh*B, hygromycin resistance cassette, and downstream of *Sdh*B (3,763-bp). **(E)** Primer pair F_ptrpC/R_hph was used to amplify the hygromycin resistance cassette (1,391-bp). **(F)** The sequence of the fragment from F_detSdhB/R_detSdhB in the strain Mont-1(SdhB^G277R^)-1 indicated the successful replacement from SdhB-277G to SdhB-277R.

Since the sensitivity of *F. brasiliense* and *F. virgulforme* strains to fluopyram was not statistically different, the amino acid difference at codon 28 of SdhB between *F. brasiliense* and *F. virguliforme* does not appear to affect their sensitivity. On the other hand, *F. tucumaniae* strains contained SdhB-277R and were significantly more sensitive to fluopyram than the strains of the other two *Fusarium* species. Therefore, the function of the amino acid difference in SdhB was characterized using a reverse genetic approach. The SdhB-277G of *F. virguliforme* strain Mont-1 was replaced with SdhB-277R using a split marker method and the target gene replacement in the two mutants was confirmed by PCR with four primer pairs (**Figure 2A**). A 2,058-bp fragment of the left flanking region and 2,171-bp fragment of right flanking region were amplified only in two SdhB^G277R^ mutants by primer sets F_detSdhB/R_YG and F_HY/R_detSdhB, respectively (**Figures 2B,C**). The fragment containing upstream and full length SdhB, hygromycin resistance cassette, and downstream *Sdh*B (3,763-bp) was amplified in two mutants by F_detSdhB/ R_detSdhB and the fragment containing only upstream and full length *Sdh*B and downstream *Sdh*B (2,372-bp) was amplified in the *F. virguliforme* strain Mont-1 (**Figure 2D**). The hygromycin resistance cassette (1,391-bp) was amplified by primer set F_ptrpC and R_hph and detected in only two mutants (**Figure 2E**). The sequence of the fragment amplified by F_detSdhB/ R_detSdhB in the mutants confirmed SdhB-277G was replaced by SdhB-277R (**Figure 2F**).

**FIGURE 3:**
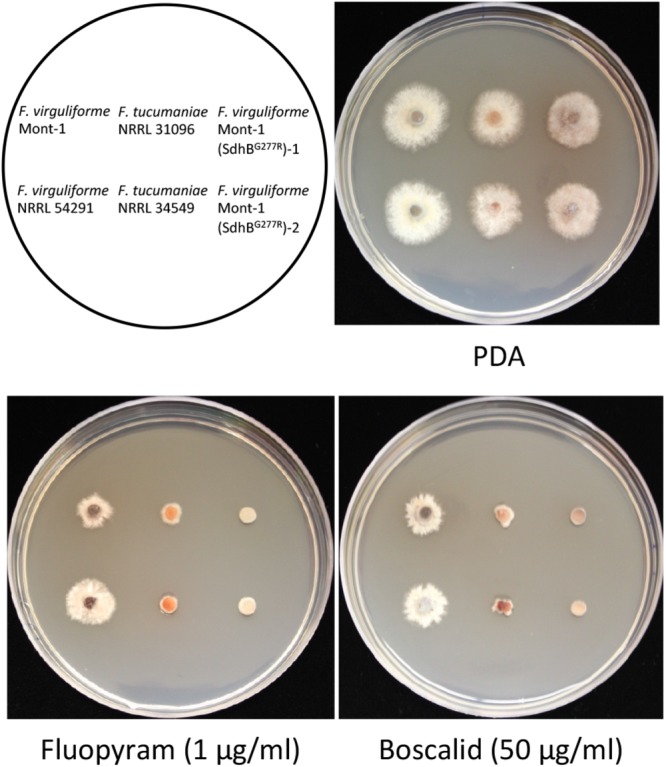
Sensitivity of two *F. virguliforme* strains (Mont-1 and NRRL 54291) and two mutants (Mont-1(SdhB^G277R^)-1 and -2) and two *F. tucumaniae* strains (NRRL 31096 and NRRL 34549) to two SDHI fungicides, fluopyram and boscalid. The pictures were taken after 5 days of growth on half strength PDA without or with fluopyram (1 μg ml^-1^) or boscalid (50 μg ml^-1^).

On half-strength PDA, there was no statistical difference in growth rate of any strains used for *in vitr*o sensitivity tests. Two mutants Mont-1(SdhB^G277R^)-1 and -2 and *F. tucumaniae* strains NRRL 31096 and NRRL 34549 were more sensitive to fluopyram (1 μg ml^-1^) and boscalid (50 μg ml^-1^) than the two wild type *F. virguliforme* strains Mont-1 and NRRL 54291. These results validate that the substitution SdhB^G277R^ in *F. virguliforme* confers increased sensitivity to two different SDHI fungicides (**Figure 3**).

### A G277R Substitution in SdhB of *F. virguliforme* Reduces Soybean SDS and Root Rot Disease in the Presence of Fluopyram

**FIGURE 4:**
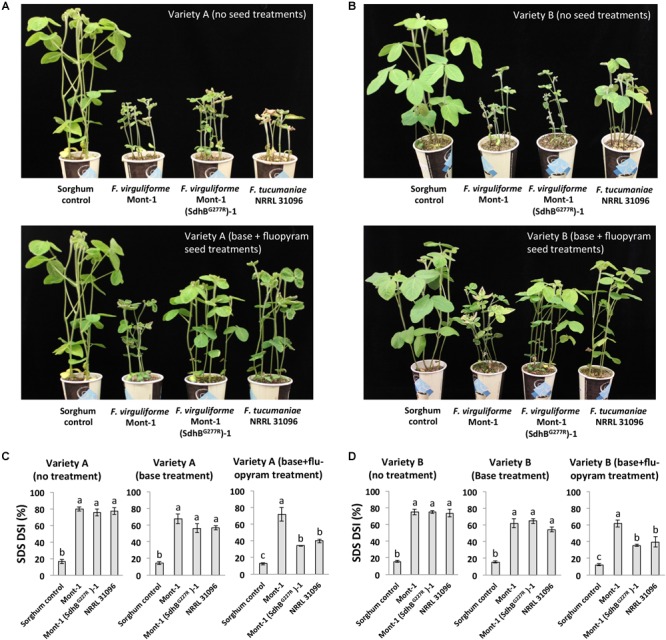
Soybean sudden death syndrome (SDS) caused by *F. virguliforme* strains Mont-1 and Mont-1(SdhB^G277R^)-1 and *F. tucumaniae* strains NRRL 31096 without and with fluopyram. **(A)** The picture of soybean SDS from variety A with no treatment and with base + fluopyram treatment infected by strains Mont-1, Mont-1(SdhB^G277R^)-1, NRRL 31096, and sorghum control 28 days after inoculation. **(B)** The picture of soybean SDS from variety B with no treatment and with base + fluopyram treatment infected by strains Mont-1, Mont-1(SdhB^G277R^)-1, NRRL 31096, and sorghum control 28 days after inoculation. **(C)** SDS disease severity index (DSI) on soybean variety A with no treatment, base treatment, and base + fluopyram treatment infected by three *Fusarium* strains. Means followed by the same letter are not significantly different (*P* < 0.05) using Fisher’s Protected Least Significant Difference test. **(D)** SDS disease severity index (DSI) on soybean variety B with no treatment, base treatment, and base + fluopyram treatment infected by three *Fusarium* strains.

To assay whether the SdhB^G277R^ substitution in *F. virguliforme* affects soybean health in seed treated with fluopyram, a soybean seedling pathogenicity assay was conducted using two soybean varieties and three different treatments: no seed treatment, a base treatment, and base + fluopyram treatment. On plants with no seed treatment, *F. virguliforme* strain Mont-1, *F. tucumaniae* NRRL 31096, and *F. virguliforme* mutant strain Mont-1(SdhB^G277R^)-1 caused interveinal chlorosis and necrosis typical symptoms of SDS (**Figures 4A,B**). Regardless of soybean variety, without seed treatment or with only a base treatment the *F. virguliforme* strains Mont-1 and Mont-1(SdhB^G277R^)-1 and *F. tucumaniae* NRRL 31096 caused significantly higher SDS disease severity index than the sorghum control. However, with the base + fluopyram treatment, *F. virguliforme* Mont-1(SdhB^G277R^)-1 and *F. tucumaniae* NRRL 31096 caused significantly less SDS symptoms on the two different varieties of soybean than the *F. virguliforme* wild type strain Mont-1 (**Figures 4C,D**).

**FIGURE 5:**
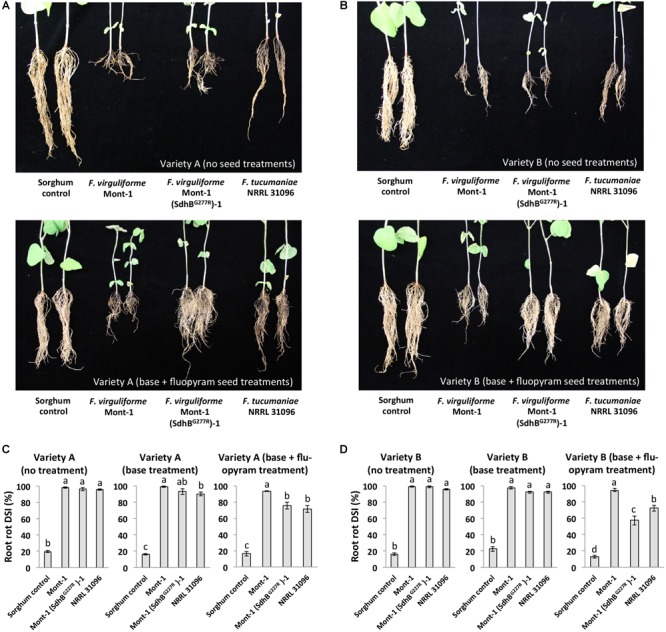
Root rot caused by *F. virguliforme* strains Mont-1 and Mont-1(SdhB^G277R^)-1 and *F. tucumaniae* strains NRRL 31096 without and with fluopyram. **(A)** The picture of soybean root rot from variety A with no treatment and with base + fluopyram treatment infected by strains Mont-1, Mont-1(SdhB^G277R^)-1, NRRL 31096, and sorghum control 28 days after inoculation. **(B)** The picture of soybean root rot from variety A with no treatment and with base + fluopyram treatment infected by strains Mont-1, Mont-1(SdhB^G277R^)-1, NRRL 31096, and sorghum control 28 days after inoculation. **(C)** Root rot disease severity index (DSI) on soybean variety A with no treatment, base treatment, and base + fluopyram treatment infected by three *Fusarium* strains. Means followed by the same letter are not significantly different (*P* < 0.05) using Fisher’s Protected Least Significant Difference test. **(D)** Root rot disease severity index (DSI) on soybean variety B with no treatment, base treatment, and base + fluopyram treatment infected by three *Fusarium* strains. Means followed by the same letter are not significantly different (*P* < 0.05) using Fisher’s Protected Least Significant Difference test.

*Fusarium virguliforme* strains Mont-1 and Mont-1(SdhB^G277R^)-1 and *F. tucumaniae* NRRL 31096 caused significant root rot disease on both varieties of soybean seedlings without a seed treatment or with only a base treatment than the sorghum control (**Figure 5**). In the base + fluopyram treatment, both varieties of soybean seedlings infected by strains *F. virguliforme* Mont-1(SdhB^G277R^)-1 and *F. tucumaniae* NRRL 31096 displayed significantly lower root rot disease than the ones infected by wild type strain *F. virguliforme* Mont-1 (**Figures 5C,D**).

Soybean seedling dry root and shoot mass (g) from both varieties of seedlings infected by *F. virguliforme* Mont-1 and Mont-1(SdhB^G277R^)-1 and *F. tucumaniae* NRRL 31096 were statistically less than seedlings grown with the sorghum control, except for seedlings from seed variety B with base + fluopyram treatment (**Tables 2, 3**). The seedlings from seed variety A with base + fluopyram treatment challenged with strains *F. virguliforme* Mont-1(SdhB^G277R^)-1 and *F. tucumaniae* NRRL 31096 had greater dry root and shoot mass than seedlings challenged with the wild type *F. virguliforme* strain Mont-1. Although the dry root and shoot mass from seed variety B with base + fluopyram was not statistically different regardless of strains used, the strains *F. virguliforme* Mont-1(SdhB^G277R^)-1 and *F. tucumaniae* NRRL 31096 resulted in a numerically greater dry root and shoot mass than the *F. virguliforme* wild type strain Mont-1 (**Tables 2, 3**).

**Table 2:** Dry root and shoot mass (g) of soybean variety A caused by *Fusarium virguliforme* strains Mont-1 and Mont-1(SdhB^G277R^)-1 and *F. tucumaniae* strain NRRL 31096.

Strain	Dry root mass (g)			Dry shoot mass (g)		
	No treatment	Base	Base + Fluopyram	No treatment	Base	Base + Fluopyram
Mont-1	0.18 ± 0.01 b*	0.25 ± 0.08 b	0.29 ± 0.04 c	0.35 ± 0.17 b	0.57 ± 0.14 b	0.59 ± 0.08 c
Mont-1(SdhB^G277R^)-1	0.19 ± 0.09 b	0.34 ± 0.14 b	0.53 ± 0.08 b	0.66 ± 0.03 b	0.81 ± 0.31 b	1.14 ± 0.16 b
NRRL 31096	0.30 ± 0.02 b	0.52 ± 0.07 b	0.59 ± 0.10 b	0.71 ± 0.11 b	0.98 ± 0.19 b	1.27 ± 0.23 b
Sorghum control	1.40 ± 0.05 a	1.15 ± 0.16 a	1.09 ± 0.03 a	3.00 ± 0.08 a	2.51 ± 0.49 a	2.35 ± 0.63 a

*^∗^Means followed by the same letter are not significantly different (*P* < 0.05) using Fisher’s Protected Least Significant Difference test.*

**Table 3:** Dry root and shoot mass (g) of soybean variety B caused by *Fusarium virguliforme* strains Mont-1 and Mont-1(SdhB^G277R^)-1 and *F. tucumaniae* strain NRRL 31096.

Strain	Dry root mass (g)			Dry shoot mass (g)		
	No treatment	Base	Base + Fluopyram	No treatment	Base	Base + Fluopyram
Mont-1	0.13 ± 0.02 b*	0.20 ± 0.04 c	0.35 ± 0.09 b	0.35 ± 0.09 b	0.50 ± 0.09 b	0.78 ± 0.06 b
Mont-1(SdhB^G277R^)-1	0.18 ± 0.04 b	0.21 ± 0.13 c	0.53 ± 0.16 ab	0.53 ± 0.10 b	0.68 ± 0.16 b	1.11 ± 0.16 ab
NRRL 31096	0.35 ± 0.12 b	0.51 ± 0.06 b	0.53 ± 0.09 ab	0.80 ± 0.23 b	0.99 ± 0.11 b	1.12 ± 0.05 ab
Sorghum control	1.07 ± 0.14 a	1.01 ± 0.10 a	1.03 ± 0.17 a	2.62 ± 0.33 a	2.55 ± 0.30 a	2.59 ± 0.56 a

*^∗^Means followed by the same letter are not significantly different (*P* < 0.05) using Fisher’s Protected Least Significant Difference test.*

## Discussion

Fluopyram as a soybean seed treatment is increasingly being implemented throughout North America to manage SDS. Our findings indicate that *F. tucumaniae* is the most sensitive species to fluopyram among the three main causal species of *Fusarium* responsible for soybean SDS. The greater sensitivity in *F. tucumaniae* was attributed to the amino acid (277R) in SdhB, which was confirmed by *F. virguliforme* SdhB^G277R^ mutants displaying increased sensitivity to fluopyram and boscalid. Significantly, the *F. virguliforme* SdhB^G277R^ mutant reduced SDS foliar symptoms and root rot severity and increased shoot and root mass in the soybean seedlings from seeds treated with fluopyram compared to a wild type *F. virguliforme* strain. These findings suggest that a single amino acid difference in the SDHI target complex between two major SDS pathogens significantly affects their sensitivity to the fungicide. This discovery is valuable to both the soybean industry and farmers in the use of fluopyram as a seed treatment, especially in South America, where *F. tucumaniae* is more prevalent than *F. virguliforme*.

*Fusarium virguliforme* and *F. brasiliense* strains displayed similar sensitivity to fluopyram. The mean EC_50_ values of *F. virguliforme* and *F. brasiliense* to fluopyram were 1.96 μg ml^-1^ and 2.21 μg ml^-1^, respectively, which are close to the baseline sensitivity of 113 *F. virguliforme* isolates conducted by Wang et al. (2017). The EC_50_ values of these 113 out of 130 isolates were lower than 5 μg ml^-1^ of fluopyram, with the remaining 17 isolates being insensitive (>5 μg ml^-1^) to fluopyram (Wang et al., 2017). The strains used in the current study were sensitive to fluopyram (lower than 5 μg ml^-1^), which were collected before registration of fluopyram or from a field location with no history of fluopyram applications. Therefore, the sensitivity of the present panel of strains could be used as a baseline sensitivity of *F. virguliforme* and *F. brasiliense* for future fungicide resistance monitoring to fluopyram in soybean fields, and also other important legume crops such as dry bean, where production is constrained by *F. brasiliense* (Jacobs et al., 2018).

The amino acid sequences of SdhB, SdhC, and SdhD from strains of three *Fusarium* species were highly conserved because the three species are evolutionarily closely related, belonging to FSSC clade 2. Although most *F. brasiliense* strains harbor the different amino acid (SdhB-28C) from strains of *F. virguliforme* and *F. tucumaniae*, the sensitivity of *F. brasiliense* and *F. virguliforme* strains to fluopyram were not statistically different and the position of SdhB-28C is not close to the quinone binding sites (Fraaije et al., 2012). Interestingly, the SdhB-277G is a conserved amino acid in this position among *Fusarium* species (**Supplementary Figure S1**) but *F. tucumaniae* is the only species containing SdhB-277R. This position is adjacent to putative SDHI fungicides’ binding sites in SdhB (Fraaije et al., 2012; Scalliet et al., 2012), thus SdhB-277R and the substitution SdhB^G277R^ might affect the binding of fluopyram to SdhB in *F. tucumaniae* and *F. virguliforme* mutants developed in this study, respectively. Some mutations (e.g., SdhB-H277Y in *Alternaria alternate* and SdhB-H267Y in *Sclerotinia homoeocarpa*) confer resistance to carboximide fungicides such as boscalid but increased sensitivity to fluopyram (Avenot and Michailides, 2010; Ishii et al., 2011; Popko et al., 2018), because fluopyram binds to a different cavity than other SDHIs (Fraaije et al., 2012). However, field and lab strains containing mutations in SdhB (e.g., P225F and H272L in *Botrytis cinerea*, H267L and N271K in *Mycosphaerella graminicola*) displayed reduced sensitivity to both carboximide and fluopyram (Scalliet et al., 2012; Veloukas et al., 2013). *F. virguliforme* SdhB^G277R^ mutants in this study exhibited increased sensitivity to both fluopyram and boscalid, suggesting that the SdhB(277R) is associated with cross sensitivity to different SDHIs. Indeed, *F. tucumaniae* strains were also more sensitive to both fluopyram and boscalid compared to *F. virguliforme* strains. To our knowledge, this is the first report for SdhB-277R as a causal genetic variant and functional verification for its novel mechanistic function involving SDHI sensitivity in fungi.

The single amino acid substitution (G277R) in SdhB of *F. virguliforme* and 277R of SdhB in *F. tucumaniae* positively affects soybean health in the presence of fluopyram. Reduced severity of SDS and root rot by fluopyram increased the amount of shoots and roots, enhancing uptake of nutrients and water in the soil. In addition, the *in vitro* sensitivity of *F. tucumaniae* and *F. brasiliense* to fluopyram indicates that this seed treatment will likely have efficacy in South America where all three species can cause SDS. In North America fluopyram has been demonstrated to reduce SDS severity and protect yields from SDS caused by *F. virguliforme* (Kandel et al., 2016, 2018). Field studies of soybean with the fluopyram seed treatment against these other *Fusarium* species (*F. tucumaniae* and *F. brasiliense*) should be conducted to evaluate efficacy of fluopyram for management of SDS under field conditions.

Fungal pathogens have quickly evolved fungicide resistance to single site mode of action fungicides when continuously exposed to selection pressure (Brent and Hollomon, 2007; Jo et al., 2008). Plant pathogenic fungi have already developed resistance to many different classes of fungicides such as methyl benzimidazole carbamates, demethylation inhibitors, quinone outside inhibitors, and SDHIs (FRAC, 2018b). Although fluopyram has been used as a soybean seed treatment and applied once per year, *F. virguliforme* isolates with insensitivity to fluopyram are already present in soybean fields and it may imply potential risk for accumulating insensitive isolates by selection pressure (Wang et al., 2017). Therefore, a robust fungicide monitoring system with large number of isolates and SDS casual *Fusarium* species from different locations is needed to prevent possible accumulation of insensitivity isolates, which might lead to disease management failure. In addition, different combinations of seed treatments should be tested in the field for a long-term management of these important fungal pathogens.

## Author Contributions

HS, AW, JJ, H-XC, and MC designed the research. HS and AW performed the experiments and analyzed the data. JJ, JW, H-XC, and MR provided input on the experiments. HS and MC wrote the manuscript. AW, JJ, JW, H-XC, and MR edited the manuscript.

## Conflict of Interest Statement

The authors declare that the research was conducted in the absence of any commercial or financial relationships that could be construed as a potential conflict of interest.
