# Supplementary material for: Fluopyram Sensitivity and Functional Characterization of SdhB in the Fusarium solani Species Complex Causing Soybean Sudden Death Syndrome
Source: Front Microbiol. 2018 Oct 1;9:2335. doi: 10.3389/fmicb.2018.02335 (PMC6174223; doi:10.3389/fmicb.2018.02335)
Supplement: Supplementary file 1 [file Table_1.pdf]

TABLE S1. Primers used in this study.

Primers name	Primer sequence (5'-3')	Description
F_SdhB	ATGGCCGCCCTCCGATC	Amplification and sequencing of *Sdh*B
R_FSdhB	TTAGTTTCCGAGAGCCATCTGC	
R_FtSdhB	TTAGTTTCTGAGGGCCATCTGC	
F_upSdhB	GGATTGAACGGCTTTTCTTC	
R_downSdhB	TATACACAGGGTTACAGTCCT	
F_SdhC	ATGCTCGCTCAACGTGTT	Amplification and sequencing of *Sdh*C
R_SdhC	TTACACAAAGGCAACCAGG	
F_upSdhC	ATACTTGGATTGGAGATCCAGT	
R_downSdhC	CTCAAAGTCAGGAATTGCCC	
F_SdhD	ATGGCCTCAATTGTGCGT	Amplification and sequencing of *Sdh*D
R_SdhD	TCATGCGTTCCAGATCCG	
F_upSdhD	AGACGTGCAATTCGAGATC	
R_downSdhD	CAGTCCTCTTGACTCGAGA	
F_KpnI_FvSdhB	ATTTGGTACCATGACGACGAGAAACCTGGA	Amplification and cloning of upstream and full length of *Sdh*B to Topo-hph plasmid
R_SpeI_FtSdhB	ATGCACTAGTTTAGTTTCTGAGAGCCATCTGCTTCT	
F_NotI_downSdhB	ATAAGCGGCCGCGAATCAAAAAGCGAATCTTT	Amplification and cloning of *Sdh*B downstream
R_NsiI_downSdhB	GCGCATGCATCGTTGAAAAGGAAGAATACAATG	
F_FvSdhB	ATGACGACGAGAAACCTGGATAC	Amplification of upstream and full length of *Sdh*B and part of hph
R_YG	CGTTGCAAGACCTGCCTGAA	
F_ HY	GGATGCCTCCGCTCGAAGTA	Amplification of *Sdh*B downstream and part of ptrpC-hph
R_downFvSdhB	CGTTGAAAAGGAAGAATACAATGTTGGC	
F_detFvSdhB	TTTCTCCCGGAACCTCATAT	To confirm transformants by PCR with R_YG or F_HY
R_detFvSdhB	TCTTAGCGGAGGTGTAGACGC	
