# Supplementary material for: Fluopyram Sensitivity and Functional Characterization of SdhB in the Fusarium solani Species Complex Causing Soybean Sudden Death Syndrome
Source: Front Microbiol. 2018 Oct 1;9:2335. doi: 10.3389/fmicb.2018.02335 (PMC6174223; doi:10.3389/fmicb.2018.02335)
Supplement: Supplementary file 3 [file Image_1.pdf]

F. verticillioides EWG46351	181	SMSLYRCHTI LNCTRACPKG LNPGKAIAEI KKQMALGN	278
F. oxysporum f.sp. lycopersici KNB08364	181	SMSLYRCHTI LNCTRACPKG LNPGKAIAEI KKQMALGN	278
F. oxysporum f.sp. vasinfectum EXM31322	181	SMSLYRCHTI LNCTRACPKG LNPGKAIAEI KKQMALGN	278
F. mangiferae CVL00991	181	SMSLYRCHTI LNCTRACPKG LNPGKAIAEI KKQMALGN	278
F. poae OBS20890	181	SMSLYRCHTI LNCTRACPKG LNPGKAIAEI KKQMALGN	278
F. graminearum CFF88817	181	SMSLYRCHTI LNCTRACPKG LNPGKAIAEI KKQMALGN	278
F. pseudograminearum EKJ73583	181	SMSLYRCHTI LNCTRACPKG LNPGKAIAEI KKQMALGN	278
F. avenaceum KIL95343	181	SMSLYRCHTI LNCTRACPKG LNPGKAIAEI KKQMALGN	278
F. virguliforme	181	SMSLYRCHTI LNCTRACPKG LNPGKAIAEI KKQMALGN	278
F. brasiliense	181	SMSLYRCHTI LNCTRACPKG LNPGKAIAEI KKQMALGN	278
F. tucumaniae	181	SMSLYRCHTI LNCTRACPKG LNPGKAIAEI KKQMALRN	278
		********** ********** ********** ****** *	

Fig. S1. Alignment of the of partial SdhB amino acid sequence from *Fusarium* species (GenBank accession no.). The shaded letter indicates difference at amino acid position 277 in *F. tucumaniae* from other species. Asterisks indicate identical amino acids among the species compared.
